# Altered neuroimaging patterns of cerebellum and cognition underlying the gait and balance dysfunction in cerebral small vessel disease

**DOI:** 10.3389/fnagi.2023.1117973

**Published:** 2023-03-01

**Authors:** Yuting Mo, Chenglu Mao, Dan Yang, Zhihong Ke, Lili Huang, Zhiyuan Yang, Ruomeng Qin, Yanan Huang, Weiping Lv, Zheqi Hu, Yun Xu

**Affiliations:** ^1^Department of Neurology, Nanjing Drum Tower Hospital Clinical College of Nanjing Medical University, Nanjing, China; ^2^Medical School, State Key Laboratory of Pharmaceutical Biotechnology, Department of Neurology, Drum Tower Hospital, Institute of Brain Science, Nanjing University, Nanjing, China; ^3^Nanjing Neurology Clinic Medical Center, Nanjing, China; ^4^Jiangsu Key Laboratory for Molecular Medicine, Medical School of Nanjing University, Nanjing, China; ^5^Jiangsu Province Stroke Center for Diagnosis and Therapy, Nanjing Drum Tower Hospital, Nanjing, China

**Keywords:** gait and balance dysfunction, cerebral small vessel disease, cognitive impairment, cortical atrophy, modular interaction

## Abstract

**Background:**

The mechanism of gait and balance dysfunction (GBD) in cerebral small vessel disease (CSVD) remains unclear. Evidence supports cognition engages in GBD of CSVD. The cerebellum is important in motor and cognition, while little is known about the influence of the cerebellum on GBD in CSVD.

**Methods:**

This study is a retrospective cohort study. All participants of this study were enrolled from the CSVD individuals in Nanjing Drum Tower Hospital from 2017 to 2021. The GBD of CSVD patients was defined as Tinetti Test score ≤ 23. Cerebral cortical thickness, cerebellar gray matter volume, the amplitude of low-frequency fluctuation, functional connectivity, and modular interaction were calculated to determine the cortical atrophy and activity patterns of CSVD patients with GBD. The effect of cognitive domains during GBD in CSVD patients was explored by correlation analyses.

**Results:**

A total of 25 CSVD patients were recruited in CSVD patients with GBD group (Tinetti Test score ≤ 23, mean age ± standard deviation: 70.000 ± 6.976 years), and 34 CSVD patients were recruited in CSVD patients without GBD group (Tinetti Test score > 23, mean age ± standard deviation: 64.029 ± 9.453 years). CSVD patients with GBD displayed worse cognitive performance and cortical atrophy in the right cerebellum VIIIa and bilateral superior temporal gyrus than those without GBD. The right postcentral gyrus, left inferior temporal gyrus, right angular gyrus, right supramarginal gyrus and right middle frontal gyrus were functionally overactivated and showed decreased modular interaction with the right cerebellum. Tinetti Test scores were negatively related to the volume of the right cerebellum VIIIa in CSVD patients with GBD. Notably, memory, especially visuospatial memory, was greatly associated with GBD in CSVD.

**Conclusion:**

The cortical atrophy and altered functional activity in sensorimotor area and ventral attention network in the cerebellum and cerebrum may underlying the GBD in CSVD. Memory might be critically cognitively responsible for GBD in CSVD.

## 1. Introduction

Cerebral small— vessel disease (CSVD), with white matter hyperintensities (WMH), lacunes, cerebral microbleeds (CMBs), perivascular space enlargement, and brain atrophy as typical neuroimaging features, has an incidence of more than 68% in the elderly over 60 years of age (Cannistraro et al., 2019; Mo et al., 2022). Gait and balance dysfunction (GBD) and cognitive impairment are two important symptoms of CSVD and closely associated with the risk of mortality in CSVD patients (van der Holst et al., 2016).

Gait and balance dysfunction can occur in CSVD patients with or without myodynamia or dystonia (van der Holst et al., 2016). Fall risk caused by GBD is highly related to disabilities and death. Tinetti Test (Tinetti, 1986), a reliable tool for the evaluation of fall risk, has the advantage of quantifying gait and balance performance, which should be a great indicator for grouping subjects to explore the mechanism of GBD in CSVD. However, there are few studies of GBD grouped according to Tinetti Test for the difficult recruitment caused by the poor abnormality of Tinetti Test in CSVD patients without myodynamia and dystonia.

The mechanism of GBD in CSVD is still unclear and the consistency of the current results is unsatisfactory. In early studies, scholars believed that WMH, lacunes, and CMBs were main reasons for gait disturbances of CSVD (de Laat et al., 2010; Li et al., 2020; Hou et al., 2021), which were contradicted by other studies (Pinter et al., 2017; Su et al., 2017). Recently, it was reported that comorbid CSVD might worsen both motor disorder and cognitive impairment in patients with Parkinson’s disease (PD) (Chen et al., 2021; Wang et al., 2022). Cognitive impairment combined with cortical changes might be a unique entry point for the study of motor decline (Jokinen et al., 2022). Motor planning, execution, and sensory function were proposed to be important for gait in the elderly (Jayakody et al., 2020). The deficit of certain cognitive domains, such as processing speed and memory, might be responsible for gait dysfunction in CSVD patients (Jokinen et al., 2022). Using analysis of structural magnetic resonance imaging (MRI) in CSVD patients, it was demonstrated that cortical thinning of frontal and temporal lobes was linked to gait disturbances (de Laat et al., 2012). However, in the existing studies of gait and cognition in CSVD patients, not only cerebellar and cerebral analyses, but also functional and structural analyses were often separated from each other.

The central nervous system is a compactly connected whole tissue, in which the cerebrum and cerebellum are structurally and functionally inseparable. The cerebellum is devoted to motor control, and also plays a critical node in the distributed neural circuits subserving behavior. The cerebellum is closely involved in cognitive function, such as linguistic function, working memory, visuospatial function, and so on (Guell et al., 2018). Moreover, multiple cerebral functional networks map onto the cerebellum, including visual, sensorimotor, dorsal attention, ventral attention, and default functions (Schmahmann et al., 2019). The atrophy of the cerebellum was found to be increasingly associated with the severity of WMH and related to memory function in WMH patients (Cao et al., 2021). However, little is currently known of the function of the cerebellum during GBD in CSVD patients. Thus, more studies of the cerebellum may provide a new breakthrough to identify the neuroregulatory pathway of GBD in CSVD.

Traditional cortical structure analysis software [such as FreeSurfer (Fischl, 2012)] performs well in the segmentation of the cerebral cortex, but not of the cerebellar cortex. A spatially unbiased atlas template of the cerebellum and brainstem (SUIT) involves the Matlab toolbox, dedicated to the analysis of imaging data of the human cerebellum, which performs well in cerebellar segmentation (Diedrichsen, 2006). In this study, we grouped subjects by Tinetti Test and focused on the altered regions and communication of the cerebral and cerebellar cortex, both structurally and functionally, using FreeSurfer, SUIT, Data Processing Assistant for Resting-State Functional MR Imaging toolkit [DPARSF (Chao-Gan and Yu-Feng, 2010)], and GRETNA (Wang et al., 2015a,b) in imaging analysis. The aims were to: (1) determine the cortical atrophy and activity patterns of CSVD patients with GBD in both the cerebrum and cerebellum; and (2) detect the effect of cognitive domains during GBD in CSVD patients.

## 2. Materials and methods

### 2.1. Participants

This study is a retrospective cohort study. The participants of this study were enrolled from the CSVD individuals in Nanjing Drum Tower Hospital from 2017 to 2021. This study was approved by the Nanjing Drum Tower Hospital Ethics Committee (Ethical Approval Code: 2017-079-04) and written informed consent was obtained from each participant.

The diagnostic criteria of CSVD are as follows: (1) lesions of moderate-to-severe WMH (Prins and Scheltens, 2015) (Fazekas scores of 2 or higher) and/or anatomically appropriate lacunes on neuroimaging, with or without enlarged perivascular spaces, microbleeds, and brain atrophy (Wardlaw et al., 2013; Lawrence et al., 2014); (2) acute symptoms (e.g., lacunar syndromes and transient ischemic attack) or subacute manifestations (e.g., cognitive impairment, motor disturbances, and emotional disorders); (3) no history of ischemic stroke with infarct size more than 1.5 cm in diameter; (4) no cerebral hemorrhage; (5) intracranial or extracranial large artery stenosis ≤ 50%.

In this study, we concentrated on the GBD, cognition and neuroimages in CSVD patients without myodynamia and dystonia. Thus, subjects with the following cases were excluded from this study: (1) left-handedness; (2) other neurological disorders, such as Alzheimer’s disease, PD, epilepsy, mental disorder, and so on; (3) any other conditions affecting gait and balance, such as limb weakness, dystonia, joint disease, and so on; (4) serious physical illnesses, such as cancer; (5) poor cooperation with MRI scans or clinical assessment, such as blindness, a deaf-mute, physical disability, and so on. Finally, in this study, we focused on the 535 CSVD patients aged from forty to eighty who underwent multimodal MRI scans as well as standardized clinical assessment in the Nanjing Drum Tower Hospital.

Tinetti Test was used to identify the GBD of CSVD patients (Tinetti Test score > 23: no disturbance in gait or balance; Tinetti Test score ≤ 23: disturbance in gait or balance). Of the 535 CSVD patients, only 25 CSVD patients scored ≤ 23 in the Tinetti Test, and the remaining 510 CSVD patients scored > 23 in the Tinetti Test. Thus, 25 CSVD patients with Tinetti Test score ≤ 23 were defined as CSVD patients with GBD (CSVD-GBD) group. Meanwhile, 34 CSVD patients, which were defined as the CSVD patients without GBD (CSVD-no-GBD) group, were randomly selected by systematic sampling with the sample distance of 15 from 510 CSVD patients with Tinetti Test scores > 23.

### 2.2. Clinical assessments

The clinical assessments included demographic data collecting, vascular risk factors recording, neuropsychological evaluation, and Tinetti Test. Tinetti Test was conducted to define CSVD-GBD and CSVD-non-GBD groups. The standardized neuropsychological test protocol contained Hamilton Depression Rating Scale (HAMD) (Hamilton, 1960) and Hamilton Anxiety Rating Scale (HAMA) (Hamilton, 1959), Minimum Mental State Examination (MMSE), Montreal Cognitive Assessment (MoCA) (Lu et al., 2011) (Beijing version 26 August 2006 translated by Wei Wang and Hengge Xie),^1^ Auditory Verbal Learning Test (AVLT) (Zhao et al., 2012, 2015), Wechsler Memory Scale-Visual Reproduction (VR), Digit Span Test (DST), Category Verbal Fluency (CVF), and Stroop Color Word Test (SCWT). The details were contained in Supplementary Information.

### 2.3. MRI data acquirement

The MRI scanning was conducted in the Nanjing Drum Tower Hospital with a Philips 3.0-T scanner (Philips Medical Systems, Netherlands). The detailed parameters were contained in Supplementary Information.

### 2.4. MRI data analysis

#### 2.4.1. Structural MRI data

The structural MRI analyses were performed by Lesion Segmentation Tool [LST, a toolbox for statistical parametric mapping software package (SPM) 12] (Schmidt et al., 2012), Voxel-based morphometry toolbox for SPM8 (VBM8),^2^ FreeSurfer (version 7.2 for Linux) (Fischl, 2012), and SUIT (a toolbox based on SPM12) [covariances in Freesurfer: age, WMH volume, total CMBs and total intracranial volume (TIV); covariances in SUIT: age, WMH volume, and total CMBs].

#### 2.4.2. Functional MRI (fMRI) data analysis

##### 2.4.2.1. Voxel-based fMRI analysis

The voxel-based fMRI analysis was performed by DPARSF (Chao-Gan and Yu-Feng, 2010), adjusted for age, WMH volume, and total CMBs. Notably, the subjects with head motion more than 3.0 mm of displacement in any direction, or 3.0 degrees of rotation in any angular dimension were excluded. First, the amplitude of low-frequency fluctuation (ALFF) (0.01–0.08 Hz) was calculated by DPARSF. Second, the brain region showing significant difference in ALFF analysis was defined as the region of interest (ROI) to perform FC analysis.

##### 2.4.2.2. Modular interaction analysis

Modular interaction analysis was performed by GRETNA. The fMRI preprocessing steps were consistent with DPARSF. Modules were defined based on the results of structural and functional analysis. Area under curve (AUC) value was defined as the strength of modular interaction and was further analyzed using the general linear model (GLM) in SPSS 16.0 (SPSS, Chicago, IL, USA), adjusted for age, WMH volume, and total CMBs.

The details of MRI data analysis were contained in Supplementary Information.

#### 2.4.3. Quality control in MRI analysis

The segmentation and normalization of both structural and functional images were visually inspected and corrected manually if necessary.

### 2.5. Statistical analysis

The demographic data was analyzed by SPSS 16.0. All the continuous variables were tested for normality before comparison. Chi-Squared test and Fisher’s exact test were used for the comparison of categorical variables. Two sample *t*-test, and Mann–Whitney test were used to compare the variations of continuous variables between CSVD-GBD group and CSVD-no-GBD group according to the normality. Additionally, demographics and CSVD neuroimaging features showing remarkable differences between CSVD-GBD group and CSVD-no-GBD group were added as covariates in GLM for the comparisons of the strength of modular interaction and cognitive scale scores which corresponded to normal distribution. The statistical analyses of MRI parameters were described in Supplementary Information. Pearson correlation, Spearman correlation and partial correlation were conducted to explore the relationship among MRI parameters, cognitive scale scores, and Tinetti Test scores. Covariates in GLM and partial correlation with non-normal distribution were transformed by logarithm. Bonferroni correction was applied in GLM for multiple comparison corrections. *P* < 0.05 was considered statistically significant.

## 3. Results

### 3.1. Clinical assessments

Demographically, there was no significant difference in sex, education year, and vascular risk factors. The age of CSVD-GBD group was older than that of CSVD-no-GBD group. In neuroimaging features, there were significant differences in WMH volume and total CMBs, while no difference in cerebellar CMBs. There was also no difference in the count of lacunes and TIV. In the neuropsychological evaluation, there was no remarkable difference in HAMA and HAMD. Patients in CSVD-GBD group had poor performance in general cognitive function (MMSE and MoCA), DST, and CVF than those in CSVD-no-GBD group, while no difference was observed in AVLT, VR, and SCWT. Detailed information regarding the clinical assessments is shown in Table 1.

**TABLE 1:** Results of clinical assessments.

	CSVD-GBD group (*n* = 25)	CSVD-no-GBD group (*n* = 34)	Statistics	*P*-value
**Demographics**				
**Sex**				
Male	14	18	0.054	0.816^a^
Female	11	16		
Age (years, mean ± s.d.)	70.000 ± 6.976	64.029 ± 9.453	2.667	0.010^c^
Education (years, mean ± s.d.)	10.500 ± 4.291	10.677 ± 4.132	−0.159	0.874^c^
Vascular risk factors				
Hypertension, *n* (%)	18 (72.000%)	19 (55.882%)	1.600	0.206^a^
Diabetes, *n* (%)	8 (32.000%)	7 (20.558%)	0.990	0.320^a^
Hyperlipidemia, *n* (%)	4 (16.000%)	5 (14.706%)		1.000^b^
Smoking, *n* (%)	4 (16.000%)	9 (26.470%)		0.526^b^
Drinking, *n* (%)	2 (8.000%)	6 (17.647%)		0.447^b^
**CSVD neuroimaging features**				
WMH volume [ml, median (interquartile range)]	13.337 (4.222, 33.670)	6.128 (2.735, 11.971)	−2.393	0.017^d^
Total lacunes [*n*, median (interquartile range)]	1.000 (0.000,4.500)	2.000 (0.000, 3.000)	−0.507	0.612^d^
Cerebellar lacunes [*n*, median (interquartile range)]	0.000 (0.000, 0.000)	0.000 (0.000, 0.000)	−1.166	0.244^d^
Total CMBs [*n*, median (interquartile range)]	3.000 (0.000, 6.000)	0.000 (0.000, 0.000)	−2.979	0.003^d^
Cerebellar CMBs [*n*, median (interquartile range)]	0.000 (0.000, 0.000)	0.000 (0.000, 0.000)	−1.184	0.236^d^
Total intracranial volume (mean ± s.d.)	1329.679 ± 118.291	1364.738 ± 115.173	−1.142	0.258^c^
Tinetti Test [median (interquartile range)]	19.000 (15.000, 22.000)	28.000 (28.000, 28.000)	−7.255	0.000^d^
**Neuropsychological data**				
HAMD (mean ± s.d.)	6.600 ± 4.481	4.706 ± 3.335	1.863	0.068^c^
HAMA (mean ± s.d.)	9.280 ± 6.736	8.471 ± 6.774	0.455	0.651^c^
MMSE [median (interquartile range)]	26.000 (23.500, 29.000)	28.500 (28.000, 29.000)	−3.045	0.002^d^
MoCA (mean ± s.e.m.)	20.440 ± 0.897	23.236 ± 0.759	5.202	0.027^e^
DSF [median (interquartile range)]	8.000 (7.000, 8.000)	8.000 (8.000, 9.000)	−2.183	0.029^d^
DSB (mean ± s.e.m.)	3.585 ± 0.204	4.344 ± 0.184	7.108	0.010^e^
CVF (mean ± s.e.m.)	13.578 ± 0.880	17.331 ± 0.775	9.713	0.003^e^
VRC [median (interquartile range)]	14.000 (13.000, 14.000)	14.000 (14.000, 14.000)	−1.374	0.169^d^
VRIR (mean ± s.e.m.)	7.937 ± 0.766	8.974 ± 0.643	1.026	0.317^e^
VRDR (mean ± s.e.m.)	6.539 ± 0.891	7.186 ± 0.747	0.296	0.589^e^
VRR (mean ± s.e.m.)	2.127 ± 0.281	2.342 ± 0.229	0.336	0.565^e^
SCWT–B (mean ± s.e.m.)	30.504 ± 2.624	26.131 ± 2.216	1.487	0.229^e^
SCWT–CB (mean ± s.e.m.)	14.265 ± 2.942	10.554 ± 2.530	0.838	0.365^e^
AVLTIR (mean ± s.e.m.)	12.090 ± 1.103	14.436 ± 0.926	2.546	0.118^e^
AVLTSTDR (mean ± s.e.m.)	2.887 ± 0.546	4.113 ± 0.446	2.914	0.095^e^
AVLTLTDR (mean ± s.e.m.)	2.548 ± 0.537	3.726 ± 0.447	2.733	0.106^e^
AVLT–recognition (mean ± s.e.m.)	18.160 ± 0.910	19.641 ± 0.744	1.532	0.223^e^

The description of statistics in general linear model analysis were after estimation. Bonferroni correction was applied in general linear model for multiple comparison corrections. CSVD, cerebral small vessel disease; GBD, gait and balance dysfunction; CSVD-GBD, CSVD patients with GBD; CSVD-no-GBD, CSVD patients without GBD; WMH, white matter hyperintensities; CMBs, cerebral microbleeds; HAMD, Hamilton Depression Rating Scale; HAMA, Hamilton Anxiety Rating Scale; MMSE, Minimum Mental State Examination; MoCA, Montreal Cognitive Assessment; DSF, Digit Span Test-forward; DSB, Digit Span Test-backward; CVF, Category Verbal Fluency; VR, Wechsler Memory Scale-Visual Reproduction; VRC, VR–copy; VRIR, VR-immediate recall; VRDR, VR-delayed recall; VRR, VR-recognition; SCWT, Stroop Color Word Test; AVLTIR: Auditory Verbal Learning Test–immediate recall; AVLTSTDR: Auditory Verbal Learning Test–short time delay recall; AVLTLTDR: Auditory Verbal Learning Test–long time delay recall; AVLT–recognition: Auditory Verbal Learning Test–recognition; s.d., standard deviation; s.e.m., standard error of mean. ^a^*P*-value of chi-squared test. ^b^*P*-value of Fisher’s Exact test. ^c^*P*-value of independent t test. ^d^*P*-value of Mann-Whitney test. ^e^*P*-value of general linear model, adjusted for age, WMH volume, and total CMBs.

### 3.2. Structural analysis

In cerebral structure, the cortical thickness in the bilateral superior temporal gyrus (STG) were thinner in CSVD-GBD group than in CSVD-no-GBD group (analyzed by Freesurfer). In cerebellar structure, there was gray matter atrophy in the right cerebellum VIIIa [Anatomical Automatic Labeling (AAL) 104, cerebellum_8_R] in CSVD-GBD group than in CSVD-no-GBD group (analyzed by SUIT). Detailed information of atrophic brain regions is shown in Table 2.

**TABLE 2:** Regions showing significant atrophy in cortex between CSVD-GBD group and CSVD-no-GBD group.

Brain region	Side	AAL	MNI coordinates			Cluster size	CSVD-GBD group* (*n* = 25)	CSVD-no-GBD group* (*n* = 34)
			*x*	*y*	*z*			
Cerebellum VIIIa^a^	right	104	24	−60	−47	121 voxels	0.844 ± 0.018	0.863 ± 0.014
Superior temporal gyrus^b^	right	82	45.0	−14.2	−10.7	694.13 mm^2	1.870 ± 0.236	2.226 ± 0.143
Superior temporal gyrus^b^	left	81	−48.3	−15.6	−5.4	571.84 mm^2	1.835 ± 0.249	2.200 ± 0.146

CSVD, cerebral small vessel disease; GBD, gait and balance dysfunction; CSVD-GBD, CSVD patients with GBD; CSVD-no-GBD, CSVD patients without GBD; AAL, Anatomical Automatic Labeling; MNI coordinates, montreal neurological institute coordinates of the peak voxel. ^a^The significant region in SUIT analysis, adjusted for age, white matter hyperintensities volume, and total cerebral microbleeds, using Gaussian random field correction for multiple comparisons (voxel level: *p* < 0.001, cluster level: *p* < 0.05). ^b^The significant regions in Freesurfer analysis, adjusted for age, white matter hyperintensities volume, total cerebral microbleeds, and total intracranial volume, using Monte Carlo simulation correction for multiple comparisons (vertex-wise/cluster-forming: *p* < 0.01, cluster-wise: *P* < 0.05). *The cortical thickness/gray matter volume of brain regions (mean ± standard deviation).

### 3.3. Functional analysis

Only 21 subjects in CSVD-GBD group and 30 subjects in CSVD-no-GBD group were recruited in functional analysis. Six subjects (three in CSVD-GBD group, three in CSVD-no-GBD group) were excluded for unqualified head motion. One subject in CSVD-no-GBD group was excluded for unsatisfactory normalization. One subject in CSVD-GBD group lack the blood oxygen on level depending sequence.

**FIGURE 1:**
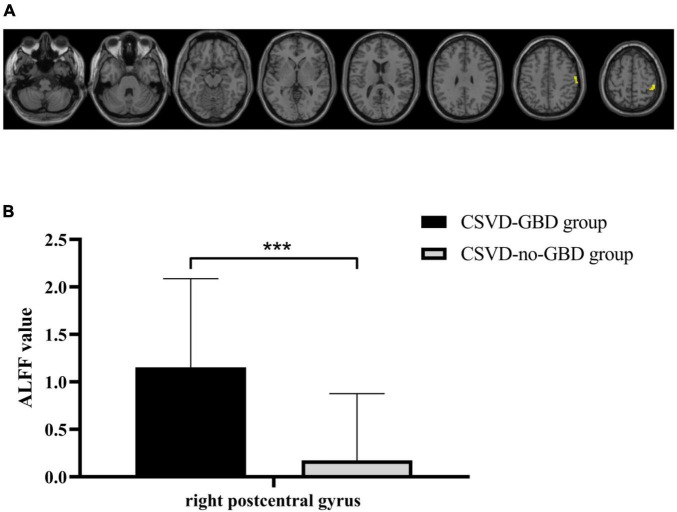
**(A)** Statistical maps of voxel *z*-values of the ALFF comparisons between CSVD-GBD group (*n* = 21) and CSVD-no-GBD group (*n* = 30). The threshold was set at *p* < 0.001 at the voxel level and *p* < 0.05 at the cluster level by Gaussian random field correction. The yellow-shaded area is the right postcentral gyrus (montreal neurological institute coordinates of the peak voxel: *x* = 48, *y* = –33, *z* = 57) which had significant increasing in ALFF. **(B)** The ALFF value of the right postcentral gyrus in CSVD-GBD group (*n* = 21) and CSVD-no-GBD group (*n* = 30) (CSVD-GBD group: 1.153 ± 0.935; CSVD-no-GBD group: 0.174 ± 0.703) (mean ± standard deviation). CSVD, cerebral small vessel disease; GBD, gait and balance dysfunction; CSVD-GBD, CSVD patients with GBD; CSVD-no-GBD, CSVD patients without GBD; ALFF, amplitude of low-frequency fluctuation. ***P < 0.001.

**FIGURE 2:**
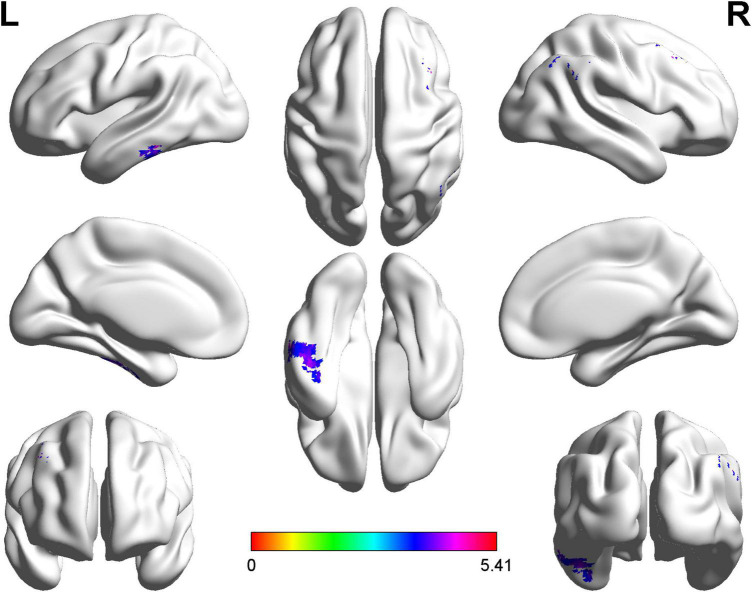
Statistical maps of voxel *z*-values of functional connectivity comparisons between cerebral small vessel disease patients with gait and balance dysfunction (CSVD-GBD) group (*n* = 21) and CSVD patients without GBD (CSVD-no-GBD) group (*n* = 30); the *z*-score bar is shown at the bottom of the map. The threshold was set at *p* < 0.001 at the voxel level and *p* < 0.05 at the cluster level by Gaussian random field correction. The purple-shaded areas correspond to the brain regions where connectivity with the right postcentral gyrus increased: the left inferior temporal gyrus, the right angular gyrus and the right supramarginal gyrus, the right middle frontal gyrus.

The right postcentral gyrus (PoCG) (AAL58) was observed remarkable increasing in ALFF after GRF-correction (Figure 1). Based on the result of ALFF analysis, the right PoCG was defined as a ROI for FC calculation. Three clusters showed strengthened FC to the right PoCG: the left inferior temporal gyrus (ITG, AAL89), the right angular gyrus (AG, AAL66) and the right supramarginal gyrus (SMG, AAL64), the right middle frontal gyrus (MFG, AAL8) (Figure 2 and Table 3).

**TABLE 3:** Regions showing significant differences in the FC of right postcentral gyrus between CSVD-GBD group and CSVD-no-GBD group.

Brain region	Side	AAL	MNI coordinate			Cluster size (voxels)	CSVD-GBD group* (*n* = 21)	CSVD-no-GBD group* (*n* = 30)
			*x*	*y*	*z*			
Angular gyrus/supramarginal gyrus	right	66/64	60	−54	36	188	0.507 ± 0.165	0.259 ± 0.149
Middle frontal gyrus	right	8	42	21	18	128	0.453 ± 0.202	0.217 ± 0.199
Inferior temporal gyrus	left	89	−66	−39	−18	242	0.517 ± 0.176	0.258 ± 0.197

CSVD, cerebral small vessel disease; GBD, gait and balance dysfunction; CSVD-GBD, CSVD patients with GBD; CSVD-no-GBD, CSVD patients without GBD; FC, functional connectivity; AAL, Anatomical Automatic Labeling; MNI coordinates, montreal neurological institute coordinates of the peak voxel. *The strength of FC (mean ± standard deviation).

**FIGURE 3:**
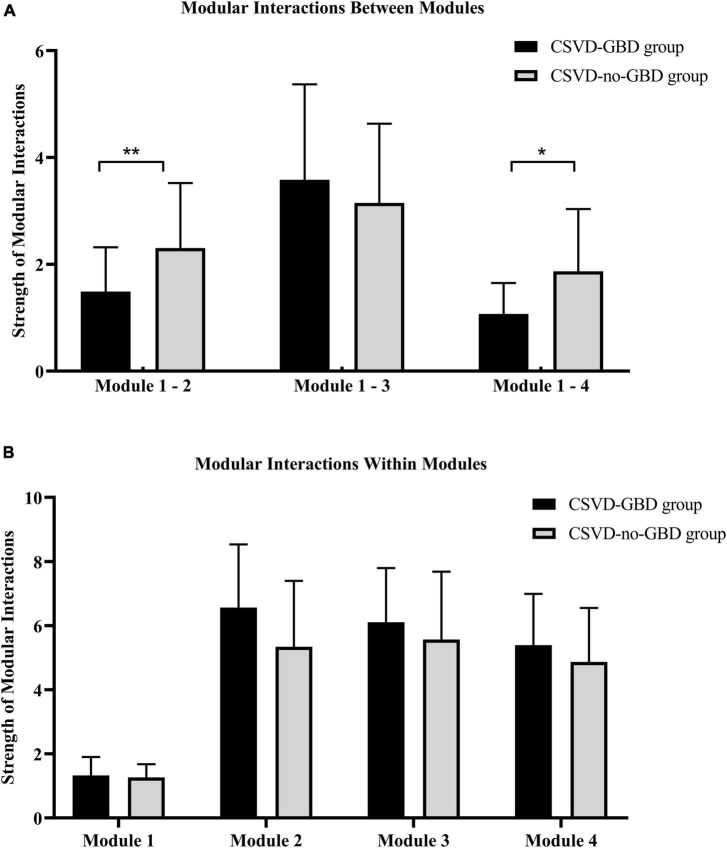
**(A)** The strength of modular interaction between modules in CSVD-GBD group and CSVD-no-GBD group. **(B)** The strength of modular interaction within modules in CSVD-GBD group and CSVD-no-GBD group. Module 1 was composed of the right postcentral gyrus, left inferior temporal gyrus, right angular gyrus, right supramarginal gyrus, and right middle frontal gyrus. Module 2 corresponded to the right cerebellar regions in the Anatomical Automatic Labeling 116 brain template. Module 3 corresponded to the left cerebellar regions in the Anatomical Automatic Labeling 116 brain template. Module 4 corresponded to the regions of vermis in the Anatomical Automatic Labeling 116 brain template. CSVD, cerebral small vessel disease; GBD, gait and balance dysfunction; CSVD-GBD, CSVD patients with GBD; CSVD-no-GBD, CSVD patients without GBD. **P < 0.01 and *P < 0.05.

Combining the results of structural analysis and functional analysis, the four modules were defined based on AAL116 brain template for modular interaction analysis. Module 1 included AAL8, AAL58, AAL64, AAL66, and AAL89. Module 2 was located in the right cerebellum, including all brain regions of the right cerebellum in AAL116 brain template (AAL92, AAL94, AAL96, AAL98, AAL100, AAL102, AAL104, AAL106, and AAL108). The left cerebellum was defined as module 3 (AAL91, AAL93, AAL95, AAL97, AAL99, AAL101, AAL103, AAL105, and AAL107). The vermis was defined as module 4 (AAL109, AAL110, AAL111, AAL112, AAL113, AAL114, AAL115, and AAL116). The strength of modular interactions between module 1 and module 2, module 1 and module 4 showed remarkable reduction in CSVD-GBD group than CSVD-no-GBD group after bonferroni correction in GLM, adjusted for age, WMH volume, and total CMBs (Figure 3A and Table 4). No significant difference was observed in the strength of modular interaction within each module (Figure 3B and Table 4).

**TABLE 4:** The results of modular interaction analysis between CSVD-GBD group and CSVD-no-GBD group.

Modular interaction	CSVD-GBD group* (*n* = 21)	CSVD-no-GBD group* (*n* = 30)	Statistics	*P*-value^a^
Between module 1 and module 2	1.369 ± 0.252	2.389 ± 0.207	8.821	0.005
Between module 1 and module 3	3.632 ± 0.371	3.115 ± 0.304	1.051	0.311
Between module 1 and module 4	1.098 ± 0.226	1.846 ± 0.185	5.910	0.019
Within module 1	1.383 ± 0.113	1.226 ± 0.093	1.036	0.314
Within module 2	6.268 ± 0.461	5.541 ± 0.378	1.342	0.253
Within module 3	5.947 ± 0.465	5.673 ± 0.381	0.188	0.667
Within module 4	5.382 ± 0.397	4.873 ± 0.326	0.888	0.351

The description of statistics in general linear model analysis were after estimation. Bonferroni correction was applied in general linear model for multiple comparison corrections. Module 1 was composed of the right postcentral gyrus, left inferior temporal gyrus, right angular gyrus, right supramarginal gyrus, and right middle frontal gyrus. Module 2 corresponded to the right cerebellar regions in the Anatomical Automatic Labeling 116 brain template. Module 3 corresponded to the left cerebellar regions in the Anatomical Automatic Labeling 116 brain template. Module 4 corresponded to the regions of vermis in the Anatomical Automatic Labeling 116 brain template. CSVD, cerebral small vessel disease; GBD, gait and balance dysfunction; CSVD-GBD, CSVD patients with GBD; CSVD-no-GBD, CSVD patients without GBD. ^a^*P*-value of general linear model, adjusted for age, white matter hyperintensities volume, and total cerebral microbleeds. *The area under curve value in modular interaction analysis (mean ± standard error of mean).

### 3.4. Correlation analysis

The cortical thickness of the STG was negatively related to the age in CSVD-GBD group (left STG: *r* = −0.472, *P* = 0.017; right STG: *r* = −0.424, *P* = 0.035) and CSVD-no-GBD group (left STG: *r* = −0.380, *P* = 0.027). However, age was not significantly related to the volume of both the right cerebellum VIIIa and Tinetti Test scores. There was a strong positive correlation between the cortical thickness of the bilateral STG in CSVD-GBD group (*r* = 0.648, *P* = 0.001) and CSVD-no-GBD group (*r* = 0.412, *P* = 0.017) after adjustion for age, while no significant relationship was observed between the gray matter volume of the right cerebellum VIIIa and the cortical thickness of the bilateral STG. Tinetti Test scores were negatively related to the gray matter volume of the right cerebellum VIIIa (*r* = −0.421, *P* = 0.036) in CSVD-GBD group.

Age, education, WMH volume, total CMBs, and total lacunes were added as covariates in the partial correlation analysis for their potential influence on cognitive function to explore the relationship between cognitive scale scores and other indicators. In CSVD-GBD group, (1) global cognitive function was closely related to the FC between the right PoCG and left ITG, the strength of modular interaction between module 1 and module 2, and the strength of modular interaction within module 1; (2) Auditory Verbal Learning Test - short time delay recall (AVLTSTDR) scores showed positive correlation to Tinetti Test scores (*r* = 0.615, *P* = 0.019); (3) VR-immediate recall (VRIR) scores were highly correlated to the cortical thickness of the left STG (*r* = −0.566, *P* = 0.028), as well as the FC between the right PoCG and the right MFG (*r* = 0.669, *P* = 0.024), right AG (*r* = 0.674, *P* = 0.023), and left ITG (*r* = 0.634, *P* = 0.036); (4) VR-delayed recall (VRDR) scores were closely correlated to ALFF in the right PoCG (*r* = 0.639, *P* = 0.034), the FC between the right PoCG and the right MFG (*r* = 0.768, *P* = 0.006), right AG (*r* = 0.727, *P* = 0.011), and left ITG (*r* = 0.878, *P* = 0.000), as well as the strength of modular interaction between module 1 and module 2 (*r* = −0.623, *P* = 0.041); (5) VR-recognition (VRR) scores were negatively associated with the cortical thickness of the bilateral STG (left STG: *r* = −0.549, *P* = 0.042; right STG: *r* = −0.693, *P* = 0.006); (6) DST - forward (DSF) scores were positively related to ALFF in the right PoCG (Spearman correlation analysis, *r* = 0.458, *P* = 0.037); (7) SCWT-CB was positively associated with the gray matter volume of the right cerebellum VIIIa (*r* = 0.510, *P* = 0.37). All these correlations were not found in CSVD-no-GBD group. Notably, the strength of modular interaction between module 1 and module 4 showed no statistically remarkable results in CSVD-GBD group in the correlation exploration. Detailed information of correlation analysis is shown in Table 5.

**TABLE 5:** Correlation coefficient between variables of interest in CSVD-GBD group.

	Tinetti Test	MMSE	MoCA	AVLTSTDR	VRIR	VRDR	VRR	DSF	SCWT-CB
Cortical thickness of the left STG					−0.566*		−0.549*		
Cortical thickness of the right STG							−0.693**		
Gray matter volume of the right cerebellum VIIIa	−0.421*								0.510*
ALFF in the right PoCG						0.639*		0.458*	
FC between the right PoCG and right MFG					0.669*	0.768**			
FC between the right PoCG and right AG					0.674*	0.727*			
FC between the right PoCG and left ITG		0.505*			0.634*	0.878***			
Modular interaction between module 1 and module 2		−0.512*				−0.623*			
Modular interaction between module 1 and module 4									
Modular interaction within module 1		0.534*	0.654**						
Tinetti Test				0.615*					

CSVD-GBD, cerebral small vessel disease patients with gait and balance dysfunction; ALFF, amplitude of low-frequency fluctuation; FC, functional connectivity; STG, superior temporal gyrus; PoCG, postcentral gyrus; MFG, middle frontal gyrus; AG, angular gyrus; ITG, inferior temporal gyrus; MMSE, Minimum Mental State Examination; MoCA, Montreal Cognitive Assessment; AVLTSTDR, Auditory Verbal Learning Test–short time delay recall; VR, Wechsler Memory Scale-Visual Reproduction; VRIR, VR - immediate recall; VRDR, VR - delayed recall; VRR, VR–recognition; DSF, Digit Span Test–forward; SCWT-CB, Stroop Color Word Test–CB. Module 1 was composed of the right postcentral gyrus, left inferior temporal gyrus, right angular gyrus, right supramarginal gyrus, and right middle frontal gyrus. Module 2 corresponded to the right cerebellar regions in the Anatomical Automatic Labeling 116 brain template. Module 4 corresponded to the regions of vermis in the Anatomical Automatic Labeling 116 brain template. *P < 0.05; **P < 0.01; ***P < 0.001.

## 4. Discussion

This study determined the cognitive and neuroimaging patterns in CSVD patients with and without GBD, conducting both structural and functional analyses in the cerebrum and cerebellum. The results mainly revealed that (1) CSVD patients with GBD had worse cognitive performance than those without GBD. (2) CSVD patients with GBD displayed cortical atrophy in the right cerebellum VIIIa and bilateral STG. (3) The module composed of the right PoCG, left ITG, right AG, right SMG, and right MFG was functionally overactivated and showed decreasing modular interactions with the right cerebellum and vermis in CSVD-GBD group than CSVD-no-GBD group. (4) The gray matter volume of the right cerebellum VIIIa and the memory function were closely correlated to GBD in CSVD patients.

This study is the first time that the right cerebellum VIIIa was reported, both structurally and functionally, to be involved during GBD in CSVD patients. Moreover, the gray matter volume of the right cerebellum VIIIa indicated medium association with Tinetti Test scores in CSVD patients with GBD. The cerebellar lobule VIII is an important part of cerebellar sensorimotor zones, and is devoted to overt movements. Lesions of the second cerebellar motor representation seldom produce obvious motor deficits, which correspond to the conditions of the CSVD patients with GBD in our study. In the Tasmanian Study of cognition and gait, the gray matter volume of the cerebellar lobule VIII was found to be involved in step length variabilities (Jayakody et al., 2021). However, the cohort of the Tasmanian Study focused on the elderly, and patients with CSVD were not considered as a separate group.

In the cerebrum, there was cortical thinning in the bilateral STG and functional overactivation in the right PoCG, right AG, right SMG, right MFG, and left ITG in CSVD patients with GBD. Cortical atrophy is one of the typical imaging manifestations of CSVD. In studies of gait disturbance in CSVD patients, cortical thinning in the temporal regions has been found to be correlated with gait parameters (de Laat et al., 2012; Kim et al., 2016). It was reported that some frontal, temporal, and parietal regions were overactivated in gait disorder patients (Zhou et al., 2020; Bayot et al., 2022). For example, PD patients with gait freezing showed higher activation in the right PoCG (Huang et al., 2021). The right PoCG, similar to the right cerebellum VIIIa, was also responsible for somatic sensory functions, which suggested that sensorimotor function might play an important role in the GBD of CSVD patients. In a previous study, stroke patients with sensorimotor deficits were more likely to experience a severe loss of postural control (Lim et al., 2022). In the elderly, overactivation of the sensorimotor network may be indicative of compensatory processes for poor gait performance (Di Scala et al., 2019). Moreover, cognitive sensorimotor exercise might provide a positive effect on gait improvement in stroke patients (Kim and Jang, 2021).

From the perspective of functional reflection from the cerebrum to cerebellum, we first found that modular interaction was decreased between the right cerebellum and the module composed of the right PoCG, left ITG, right AG, right SMG, and right MFG in CSVD patients with GBD. There are some theoretical supports for this result. First, the cerebellar lobule VIII is part of second cerebellar motor representation, which often targets regions surrounding the primary motor cerebral cortex (Schmahmann et al., 2019). Second, the right PoCG, left ITG, right AG, right SMG, and right MFG are all located in or around the temporoparietal junction (TPJ). The TPJ is an important component of the ventral attention network (VAN). Coincidently, in cerebellar resting-state maps, the right cerebellum VIIIa is also involved in the VAN. Moreover, the results of modular interaction in our study coincided with the right lateralized tendencies of the VAN. The VAN was thought to be mobilized in the gait tasks in patients with gait freezing in PD patients (Shine et al., 2013). In a study of fall risks in patients with WMH, disruption of the VAN and sensorimotor function were also found positively related to Foam Sway test scores (Crockett et al., 2022). Altogether, our results suggested the alterations of regions involving in sensorimotor function and the VAN might be critical to GBD in CSVD patients, which may provide specific inspiration for us to find the neural circuit and intervention target of CSVD gait disorder. Additionally, the impairment of vermis is responsible for imbalance and increased stride width, which are also characteristics of GBD in CSVD patients and might be the reason for reduced modular interaction between the vermis and the module composed of the right PoCG, left ITG, right AG, right SMG, and right MFG in CSVD patients with GBD.

Cognition was proposed to mediate the association between gait parameters and structural network efficiency in CSVD (Cai et al., 2021). In our study, CSVD patients with GBD showed worse cognitive performance than those without GBD. Among them, memory indicated strong associations with Tinetti Test scores and neuroimaging parameters in CSVD patients with GBD. The contribution of memory for gait has been reported in several studies, such as the Helsinki Small Vessel Disease Study, the Tasmanian study, and so on (Jayakody et al., 2019; Jokinen et al., 2022). Notably, the indexes of memory in our study, relating to GBD in CSVD patients, had two characteristics. First, visuospatial memory showed extreme engagement in GBD. Noteworthily, apart from TPJ-related regions, which have been proven to be involved in memory (Afthinos et al., 2022; Doricchi, 2022), the cortical thickness of the bilateral STG was also closely correlated with visuospatial memory. In a study of concomitant exotropia patients, decreased activation in the right STG was observed (Jin et al., 2022). In addition, increased intra-network FC in the bilateral PoCG of the SMN and decreased activation in the right cerebellum VIII were also reported, suggesting that the PoCG and the right cerebellum VIII played important roles in visual function (Jin et al., 2022). Actually, visuospatial function was related to gait indicators (Jayakody et al., 2021) and visual feedback balance training could be effective on gait disturbance in CSVD patients (Zhao et al., 2019). Similarly, Micol Avenali reported that visual cues improved the motor performance of a patient with a 10-year history of PD (Avenali et al., 2022). Second, short-term memory was directly related to GBD in CSVD patients. In the remarkable cognitive indicators of correlation analyses, AVLTSTDR, VRIR, and DSF suggested their involvements in short-term memory. Notably, AVLTSTDR scores were highly correlated with Tinetti Test scores in CSVD patients with GBD. Analogously, in a study of community-dwelling, short-term memory also suggested independent correlations with gait speeds in the population over 50 years of age (Killane et al., 2014).

Moreover, the atrophy of the bilateral STG was closely associated with each other and had an inseparable relationship with aging. In the Radboud University Nijmegen Diffusion tensor and MRI Cohort study, the age-related global reduction in cortical thickness, including the superior temporal lobe, primary visual cortex, and so on, was regarded as the reason for reduction in memory and executive functions in CSVD. However, atrophy of the right cerebellum VIIIa had no relationship with atrophy of the bilateral STG and aging, which might suggest that atrophy of the right cerebellum VIIIa was specific to high fall risks in CSVD. There was also an association between gray matter volume of the right cerebellum VIIIa and executive function. As mentioned above, the right cerebellum had reflections on frontal regions, which might be the reason for the correlation between right cerebellum VIIIa and executive function. However, the influence of cerebellar atrophy on executive function needs to be further studied.

Several limitations of the present study should be considered. First, our sample size was insufficient. Second, the detailed gait indicators, such as gait speed, stride length, and so on, were not included in the study. Third, our study used cross-sectional data and could not reveal causal effects of brain alterations during GBD in CSVD patients. In future studies, we will expand the sample size, use advanced gait assessment instruments such as the computerized detectors, and conduct long-term follow-up studies to more thoroughly describe features and mechanisms of GBD in patients with CSVD.

In conclusion, atrophy of the right cerebellum VIIIa and altered functional communication of the ventral attention network between the cerebrum and cerebellum might have been responsible for the high fall risks in CSVD patients. Sensorimotor and cognitive functions might have jointly been involved during GBD in CSVD patients. In addition, visuospatial memory and short-term memory might have both played important roles in maintaining gait and balance in CSVD patients. Memory might therefore be a critical cognitive domain responsible for GBD in CSVD patients. These results may bring us a new perspective to explore the neurological intervention target and the possibility of cognitive related therapy on GBD in CSVD patients.

## Data availability statement

The original contributions presented in this study are included in this article/Supplementary material, further inquiries can be directed to the corresponding author.

## Ethics statement

The studies involving human participants were reviewed and approved by the Nanjing Drum Tower Hospital Ethics Committee (Ethical Approval Code: 2017-079-04). The patients/participants provided their written informed consent to participate in this study.

## Author contributions

YX and YM: conceptualization. YM, CM, DY, ZK, ZY, RQ, and ZH: investigation. YM, CM, LH, YH, and WL: formal analysis. YM and CM: writing—original draft. YM, CM, and YX: writing—review and editing. All authors contributed to the article and approved the submitted version.

## Footnotes

1
www.mocatest.org

2
www.fil.ion.ucl.ac.uk/spm
