# Supplementary material for: Altered neuroimaging patterns of cerebellum and cognition underlying the gait and balance dysfunction in cerebral small vessel disease
Source: Front Aging Neurosci. 2023 Mar 1;15:1117973. doi: 10.3389/fnagi.2023.1117973 (PMC10032207; doi:10.3389/fnagi.2023.1117973)
Supplement: Supplementary file 1 [file Data_Sheet_1.docx]

Supplementary Material

Altered Neuroimaging Patterns of Cerebellum and Cognition Underlying the Gait and Balance Dysfunction in Cerebral Small Vessel Disease

**Yuting Mo^#^, Chenglu Mao^#^, Dan Yang, Zhihong Ke, Lili Huang, Zhiyuan Yang, Ruomeng Qin, Yanan Huang, Weiping Lv, Zheqi Hu, Yun Xu^*^**

^#^ These authors contributed equally to this work.

*** Correspondence:** Yun Xu: xuyun20042001@aliyun.com

**Methods**

1. Clinical assessments

Apart from demographic data collecting, vascular risk factors recording, and Tinetti Test, all the subjects underwent the standardized neuropsychological test protocol, including Hamilton Depression Rating Scale (HAMD)(Hamilton, 1960) and Hamilton Anxiety Rating Scale (HAMA)(Hamilton, 1959) which evaluate the mental statements, Minimum Mental State Examination (MMSE), Montreal Cognitive Assessment (MoCA)(Lu et al., 2011) (Beijing version 26 August 2006 translated by Wei Wang & Hengge Xie, www.mocatest.org), Auditory Verbal Learning Test (AVLT)(Zhao et al., 2015, Zhao et al., 2012), Wechsler Memory Scale-Visual Reproduction (VR), Digit Span Test (DST), Category Verbal Fluency (CVF), and Stroop Color Word Test (SCWT). Global cognition was assessed by MMSE and MoCA. AVLT, VR, DST, CVF, and SCWT were used to conduct a detailed cognitive assessment involving multiple cognitive domains. AVLT is a classic test for verbal memory and contains four main indicators: AVLT - immediate recall (AVLTIR), AVLT - short time delay recall (AVLTSTDR), AVLT - long time delay recall (AVLTLTDR), and AVLT - recognition (AVLT - recognition). To make the memory evaluation more comprehensive, the VR was also conducted for the assessment of visuospatial memory and the scores of VR - copy (VRC), VR - immediate recall (VRIR), VR - delayed recall (VRDR), and VR - recognition (VRR) were statistically analyzed. DST is a simple method for memory and attention. There are two parts to the DST: DST - forward (DSF) and DST-backward (DSB). Linguistic function was evaluated by CVF. SCWT - B was chosen to evaluate processing speed, and SCWT - CB (SCWT - CB = SCWT - C - SCWT - B) was used to assess executive function.

1. Magnetic Resonance Imaging (MRI) data acquirement

The MRI scanning was conducted in the Nanjing Drum Tower Hospital with a Philips 3.0-T scanner (Philips Medical Systems, Netherlands). The multimodal MRI scans included 1) the high-resolution T1-weighted turbo gradient echo sequence, with repetition time (TR) = 9.8 ms, flip angle (FA) = 8^◦^, echo time (TE) = 4.6 ms, field of view (FOV) = 250 mm × 250 mm, number of slices = 192, acquisition matrix = 256 × 256, thickness = 1.0 mm, 2) the fluid attenuated inversion recovery (FLAIR) sequence, with TR = 4,500 ms, TE = 333 ms, time interval (TI) = 1,600 ms, number of slices = 200, voxel size = 0.95 mm × 0.95 mm × 0.95 mm, acquisition matrix = 270 × 260, and 3) the blood oxygen on level depending (BOLD) sequence, TR = 2000ms, TE = 30 ms, number of slices = 35, voxel size = 3.00 mm × 3.00 mm × 4.00 mm, acquisition matrix = 64 × 62.

1. MRI data analysis
   1. Structural MRI data

3.1.1 Quantification of white matter hyperintensities (WMH) and total intracranial volume (TIV)

The lesions of white matter were quantified by Lesion Segmentation Tool (LST)(Schmidt et al., 2012), which is a toolbox based on statistical parametric mapping software package (SPM; [www.fil.ion.ucl.ac.uk/spm)](http://www.fil.ion.ucl.ac.uk/spm)) 12, based on T2-weighted FLAIR sequence and T1-weighted sequence. Detailed parameters are as follows: initial threshold: 0.15, parameter for the Markov Random Field: 1, maximum iterations: 50. The result of LST was defined as the value of WMH volume. Voxel-based morphometry toolbox for SPM8 (VBM8), a toolbox based on SPM8, was used to quantify the volume of gray matter, white matter, and cerebrospinal fluid. TIV was the sum of the volume of gray matter, white matter, and cerebrospinal fluid.

3.1.2 Cerebral cortical thickness analysis

FreeSurfer (version 7.2 for Linux) (Fischl, 2012) was used to perform cerebral cortical analysis, based on the high-resolution T1-weighted turbo gradient echo sequence. The pre-processing steps were as follows: 1) motion correction and conform; 2) non-uniform intensity normalization; 3) Talairach transformation; 4) intensity normalization(Sled et al., 1998); 5) stripping skull; 6) automatic subcortical segmentation of the white matter and the deep gray matter volumetric structures; 7) segmentation of the brain tissue, included gray matter (GM), white matter (WM), and cerebrospinal fluid (CSF); 8) automated topology fixer; 9) surface modeling for the GM/WM and GM/CSF boundaries(Dale et al., 1999, Fischl et al., 2001); 10) surface inflation(Fischl et al., 1999a); 11) registration to average surface space(Fischl et al., 1999b); 12) smoothing with a 10-mm full-width half-maximum (FWHM) Gaussian spatial kernel for noise reduction. The cortical thickness was calculated as the closest distance from the inner surface to the outer surface at each vertex(Fischl and Dale, 2000). The Desikan–Killiany (DK) atlas was chosen for consistent brain measurements(Klein and Tourville, 2012), as the boundaries in it are suitable for the FreeSurfer classifier.

Glmfit (analytic software in FreeSurfer) was used to analyze the whole-brain vertex-wise surface-based cortical thickness. A general linear model (GLM) was built by a different offset same slop method. Because of the participants’ diversity demographic characteristics, age, WMH volume, total cerebral microbleeds (CMBs) and TIV were served as the covariances for the GLM analysis. Multiple comparison corrections were performed using Monte Carlo simulation correction a vertex-wise/ cluster-forming threshold of 2 (P < 0.01) and a cluster-wise P < 0.05. The cortical thickness of significant brain regions was extracted for further analysis.

3.1.3 Cerebellar cortical structure analysis

A spatially unbiased atlas template of the cerebellum and brainstem (SUIT), a toolbox based on SPM12, was applied to cerebellar cortical structure analysis, based on the high-resolution T1-weighted turbo gradient echo sequence. The detailed processing steps were as follows: 1) cerebellar isolation and segmentation; 2) normalizing an individual cerebellum into the SUIT atlas template by Diffeomorphic Anatomical Registration Through Exponentiated Lie algebra (DARTEL); 3) reslicing images into SUIT space using DARTEL; 4) smoothing images with a 3-mm FWHM Gaussian spatial kernel. The volume of cerebellar region showing significant difference in two sample t-test (covariances: age, WMH volume, and TIV; multiple comparison corrections: Gaussian random field (GRF)-corrected threshold of p < 0.001 at the voxel level and p < 0.05 at the cluster level) was extracted for further analysis by Data Processing Assistant for Resting-State Functional MR Imaging toolkit (DPARSF).

1. Functional MRI (fMRI) data analysis
   1. Voxel-based fMRI analysis
      1. Preprocessing of fMRI

The fMRI analysis was based on the BOLD sequence. DPARSF(Chao-Gan and Yu-Feng, 2010), a method based on the Resting-State Functional MR Imaging Toolkit (http://www.restfmri.net) and SPM12, was used for fMRI data analysis. The preprocessing steps included removing the first ten volumes of data, slice timing correction, realignment (note that the subjects with head motion more than 3.0 mm of displacement in any direction, or 3.0 degrees of rotation in any angular dimension were excluded), reorientation, co-registration of T1 images to functional images; image segmentation for the high-resolution T1-weighted turbo gradient echo sequence and the BOLD sequence by DARTEL, nuisance covariates regression (including linear detrending, Friston 24 head motion parameters, white matter signal, cerebrospinal fluid signal, and global signal), normalization using a 12- parameter nonlinear transformation to the standard Montreal Neurological Institute (MNI) space (3 × 3 × 3 mm^3^), and smoothing functional images with a 6-mm FWHM Gaussian spatial kernel. The amplitude of low-frequency fluctuation (ALFF) analysis and functional connectivity (FC) analysis were all based on the fMRI after preprocessing.

- - 1. ALFF analysis

ALFF reveals BOLD signal strength of regional spontaneous activity(Zang et al., 2007). ALFF (0.01-0.08 Hz) was calculated by DPARSF, based on the functional images after smoothing, and was Z-transformed before statistical analysis. Two sample t-test of ALFF between cerebral small vessel disease (CSVD) patients with gait and balance dysfunction (GBD) (CSVD-GBD) group and CSVD patients without GBD (CSVD-no-GBD) group was conducted by DPARSF (multiple comparison corrections: GRF-corrected threshold of p < 0.001 at the voxel level and p < 0.05 at the cluster level), adding age, WMH volume, and total CMBs as the covariances. The significant ALFF values were extracted for further analysis.

- - 1. FC analysis

Before FC analysis, band-pass filtering (0.01-0.08Hz) was performed. The brain region showing the remarkable difference in ALFF analysis was defined as a region of interest (ROI) in FC analysis. The mean time series of the ROI was extracted, and the strengths of FC were calculated as Pearson's correlations between the averaged time series of the ROI and voxels in the remaining brain regions. Then, Pearson's correlation coefficients were normalized to a z-score matrix by using a Fisher r-to-z transformation, and the FC map was created between the ROI and the remaining brain regions. Two sample t-test of FC between CSVD-GBD group and CSVD-no-GBD group was implemented by DPARSF (multiple comparison corrections: GRF-corrected threshold of p < 0.001 at the voxel level and p < 0.05 at the cluster level), adjusted for age, WMH volume, and total CMBs. The FC values of significant brain regions were extracted for correlation analysis.

- 1. Functional network analysis

The main indicator of functional network analysis was modular interaction. Functional network analysis was performed by GRETNA(Wang et al., 2015b, Wang et al., 2015a). The fMRI preprocessing steps were consistent with DPARSF. The whole brain was divided into 116 ROIs by Anatomical Automatic Labeling (AAL) 116 atlas. The ROIs were defined as network nodes, and Pearson's correlations between the averaged time series of 116 brain regions were defined as network edges. The individual binary network was constructed, the network sparsity of which was set to: started at 0.05, stepped at 0.01, and ended at 0.4.

Modules were defined based on the results of structural analysis and functional analysis in the modular interaction analysis. The strength of modular interaction was measured by the number of network edges between and within modules. Area under curve (AUC) was calculated for statistical analysis by Matlab 2013b, based on the receiver operating characteristic (ROC) curve delineated by plotting 1-specificity and sensitivity at different thresholds of sparsity. AUC value was defined as the strength of modular interaction and was further analyzed using the GLM in SPSS 16.0 (SPSS, Chicago, IL, USA).

**References**

CHAO-GAN, Y. & YU-FENG, Z. 2010. DPARSF: A MATLAB Toolbox for "Pipeline" Data Analysis of Resting-State fMRI. *Front Syst Neurosci,* 4**,** 13.

DALE, A. M., FISCHL, B. & SERENO, M. I. 1999. Cortical surface-based analysis. I. Segmentation and surface reconstruction. *Neuroimage,* 9**,** 179-94.

FISCHL, B. 2012. FreeSurfer. *Neuroimage,* 62**,** 774-81.

FISCHL, B. & DALE, A. M. 2000. Measuring the thickness of the human cerebral cortex from magnetic resonance images. *Proc Natl Acad Sci U S A,* 97**,** 11050-5.

FISCHL, B., LIU, A. & DALE, A. M. 2001. Automated manifold surgery: constructing geometrically accurate and topologically correct models of the human cerebral cortex. *IEEE Trans Med Imaging,* 20**,** 70-80.

FISCHL, B., SERENO, M. I. & DALE, A. M. 1999a. Cortical surface-based analysis. II: Inflation, flattening, and a surface-based coordinate system. *Neuroimage,* 9**,** 195-207.

FISCHL, B., SERENO, M. I., TOOTELL, R. B. & DALE, A. M. 1999b. High-resolution intersubject averaging and a coordinate system for the cortical surface. *Hum Brain Mapp,* 8**,** 272-84.

HAMILTON, M. 1959. The assessment of anxiety states by rating. *Br J Med Psychol,* 32**,** 50-5.

HAMILTON, M. 1960. A rating scale for depression. *J Neurol Neurosurg Psychiatry,* 23**,** 56-62.

KLEIN, A. & TOURVILLE, J. 2012. 101 labeled brain images and a consistent human cortical labeling protocol. *Front Neurosci,* 6**,** 171.

LU, J., LI, D., LI, F., ZHOU, A., WANG, F., ZUO, X., JIA, X. F., SONG, H. & JIA, J. 2011. Montreal cognitive assessment in detecting cognitive impairment in Chinese elderly individuals: a population-based study. *J Geriatr Psychiatry Neurol,* 24**,** 184-90.

SCHMIDT, P., GASER, C., ARSIC, M., BUCK, D., FORSCHLER, A., BERTHELE, A., HOSHI, M., ILG, R., SCHMID, V. J., ZIMMER, C., HEMMER, B. & MUHLAU, M. 2012. An automated tool for detection of FLAIR-hyperintense white-matter lesions in Multiple Sclerosis. *Neuroimage,* 59**,** 3774-83.

SLED, J. G., ZIJDENBOS, A. P. & EVANS, A. C. 1998. A nonparametric method for automatic correction of intensity nonuniformity in MRI data. *IEEE Trans Med Imaging,* 17**,** 87-97.

WANG, J., WANG, X., HE, Y., YU, X., WANG, H. & HE, Y. 2015a. Apolipoprotein E epsilon4 modulates functional brain connectome in Alzheimer's disease. *Hum Brain Mapp,* 36**,** 1828-46.

WANG, J., WANG, X., XIA, M., LIAO, X., EVANS, A. & HE, Y. 2015b. GRETNA: a graph theoretical network analysis toolbox for imaging connectomics. *Front Hum Neurosci,* 9**,** 386.

ZANG, Y. F., HE, Y., ZHU, C. Z., CAO, Q. J., SUI, M. Q., LIANG, M., TIAN, L. X., JIANG, T. Z. & WANG, Y. F. 2007. Altered baseline brain activity in children with ADHD revealed by resting-state functional MRI. *Brain Dev,* 29**,** 83-91.

ZHAO, Q., GUO, Q., LIANG, X., CHEN, M., ZHOU, Y., DING, D. & HONG, Z. 2015. Auditory Verbal Learning Test is Superior to Rey-Osterrieth Complex Figure Memory for Predicting Mild Cognitive Impairment to Alzheimer's Disease. *Curr Alzheimer Res,* 12**,** 520-6.

ZHAO, Q., LV, Y., ZHOU, Y., HONG, Z. & GUO, Q. 2012. Short-term delayed recall of auditory verbal learning test is equivalent to long-term delayed recall for identifying amnestic mild cognitive impairment. *PLoS One,* 7**,** e51157.
